# Ultrasound-Guided Microbubble-Mediated Locoregional Delivery of Multiple MicroRNAs Improves Chemotherapy in Hepatocellular Carcinoma

**DOI:** 10.7150/ntno.63320

**Published:** 2022-01-01

**Authors:** Huaijun Wang, Zhongqian Hu, Uday Kumar Sukumar, Rajendran JC Bose, Arsenii Telichko, Jeremy J Dahl, Ramasamy Paulmurugan

**Affiliations:** Department of Radiology, Stanford University, School of Medicine, Stanford, California, USA.

**Keywords:** drug delivery, microRNA-100/microRNA-122/microRNA-10/microRNA-21, ultrasound, microbubble, hepatocellular carcinoma (HCC)

## Abstract

**Rationale:** To assess treatment effects of 4 complementary miRNAs (miRNA-100/miRNA-122/antimiRNA-10b/antimiRNA-21) encapsulated in a biodegradable PLGA-PEG nanoparticle, administered by an ultrasound-guided microbubble-mediated targeted delivery (UGMMTD) approach in mouse models of hepatocellular carcinoma (HCC).

**Methods:** *In vitro* apoptotic index was measured in HepG2 and Hepa1-6 HCC cells treated with various combinations of the 4 miRNAs with doxorubicin. Three promising combinations were further tested *in vivo* by using UGMMTD. 63 HepG2 xenografts in mice were randomized into: group 1, miRNA-122/antimiRNA-10b/antimiRNA-21/US/doxorubicin; group 2, miRNA-100/miRNA-122/antimiRNA-10b/antimiRNA-21/US/doxorubicin; group 3, miRNA-100/miRNA-122/antimiRNA-10b/US/doxorubicin; group 4, miRNA-122/anitmiRNA-10b/antimiRNA-21/doxorubicin; group 5, miRNA-100/miRNA-122/antimiRNA-10b/antimiRNA-21/doxorubicin; group 6, miRNA-100/miRNA-122/antimiRNA-10b/doxorubicin; group 7, doxorubicin only treatment; and group 8, without any treatment. Tumor volumes were measured through 18 days. H&E staining, TUNEL assay, and qRT-PCR quantification for delivered miRNAs were performed.

**Results:** *In vivo* results showed that UGMMTD of miRNAs with doxorubicin in groups 1-3 significantly (P<0.05) delayed tumor growth compared to control without any treatment, and doxorubicin only from day 7 to 18. On qRT-PCR, levels of delivered miRNAs were significantly (P<0.05) higher in groups 1-3 upon UGMMTD treatment compared to controls. TUNEL assay showed that upon UGMMTD, significantly higher levels of apoptotic cell populations were observed in groups 1-3 compared to controls. Toxicity was not observed in various organs of different groups.

**Conclusions:** UGMMTD of miRNA-100/miRNA-122/antimiRNA-10b/antimiRNA-21 combination improved therapeutic outcome of doxorubicin chemotherapy in mouse models of HCC by substantial inhibition of tumor growth and significant increase in apoptotic index.

## Introduction

Hepatocellular carcinoma (HCC) is the fourth leading cause of cancer-related death in the world 1. Although the incidence and mortality are declining in traditionally high-risk regions such as East Asia, it is still a fast-growing problem in the United States and Europe 1, 2. HCC is often diagnosed at advanced stages, so that only a small portion of patients diagnosed at early stage can benefit from curative treatments like hepatic resection, liver transplantation, and local percutaneous tumor ablation 1-4. The majority of patients need to undergo palliative treatments. First-line palliative treatment options include transarterial chemoembolization (TACE), transarterial radio-embolization (TARE), and systemic chemotherapies 2, 4. TACE utilizes locally delivered chemotherapeutic drugs to the tumor, while this approach is contraindicated in patients with decompensated liver cirrhosis due to the risk of fulminant liver failure from low liver reserve 4. In addition, drug resistance is a common issue in HCC patients with local or systemic chemotherapy, e.g., resistance to doxorubicin. Due to alterations in multiple signaling pathways and the complex pathophysiology of HCC, the treatment benefits are limited. Therefore, therapeutic strategies targeting the molecular signaling pathways of HCC are critically needed to treat patients with liver cancer of all stages.

MicroRNAs (miRNAs) are small noncoding RNAs that play crucial role in almost all main cellular pathways 5, 6. Targeted delivery of miRNAs has appeared as an efficient strategy for blocking key cellular processes involved in HCC development and progression 5-8. Among these miRNAs, miRNA-122 and antimiRNA-21 are broadly explored for their therapeutic efficacy 9-15.

**Figure 1:**
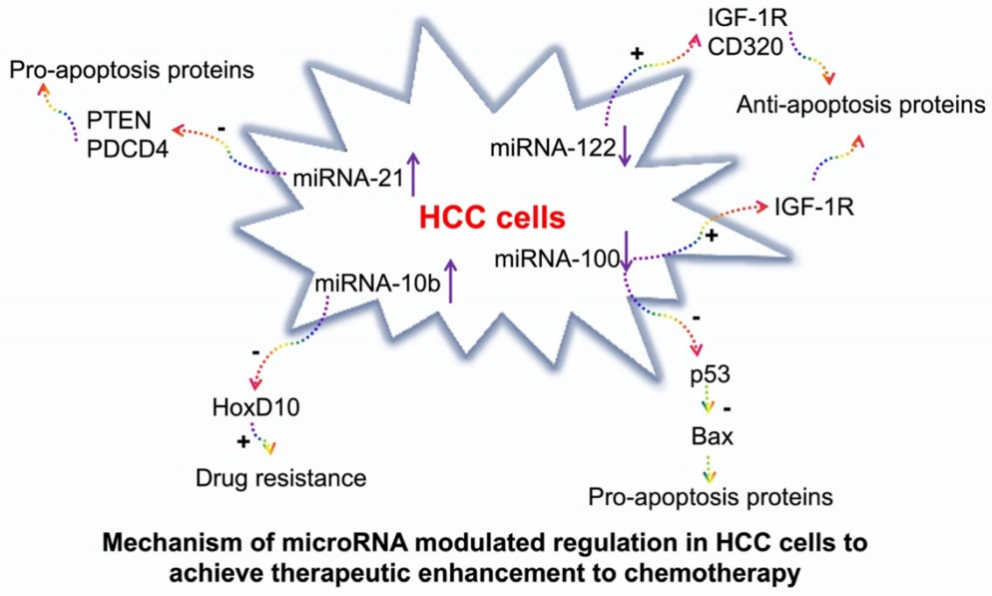
Schematic illustration of signaling pathways regulated by miRNA-100, miRNA-122, miRNA-10b, and miRNA-21 in hepatocellular carcinoma (HCC), and the mechanisms by which the microRNAs modulated regulation can achieve therapeutic enhancement of chemotherapy.

In the current study, we evaluated the treatment efficacy of 4 complementary miRNAs which are involved in the signaling pathways in producing pro- or anti-apoptosis and chemoresistance proteins (**Figure 1**). miRNA-122 induces cellular apoptosis to suppress tumor progression. It also reduces the cancer resistance to chemotherapeutic drugs, such as doxorubicin and sorafenib via the downregulation of multidrug resistance genes 12, 13. miRNA-100 inhibits carcinogenesis and proliferation of tumor cells by regulating p53 and PLK1 pathways 16, 17. In HCC, both miRNA-122 and miRNA-100 are downregulated 11. Therefore, our approach was to deliver the synthetic mimics of both the miRNAs to suppress tumor development. On the contrary, miRNA-21 plays an important role in tumor initiation, progression, and chemoresistance 14, 15. Similarly, miRNA-10b promotes tumor invasion and metastasis 18, 19. Both miRNA-21 and miRNA-10b are upregulated in HCC 11. Our approach was to deliver antisense microRNAs (antimiRNA-21 and antimiRNA-10b) targeting both miRNA-21 and miRNA-10b to inhibit their function and regulate tumor development and improve tumors response to doxorubicin.

It has recently been shown that ultrasound (US)-guided and microbubble (MB)-mediated delivery of two complementary miRNAs (miRNA-122 and antimiRNA-21) encapsulated in biodegradable poly lactic-co-glycolic acid (PLGA) - poly ethylene glycol (PEG) nanoparticles (PLGA-PEG-NPs), in combination with doxorubicin as a highly effective approach to treat resistant HCC while reducing doxorubicin doses needed for treating non-resistant HCC in longitudinal treatment experiments 9, 10. In this approach, MB-mediated cavitation inducing vascular endothelial hyperpermeability has been utilized to enhance the delivery of miRNAs from intravascular space into extracellular space, thus leading to enhanced treatment efficacy locally in the tumor 9-11, 20-23. The FDA-approved PLGA-PEG-NPs were used as the non-toxic nanocarrier for miRNAs delivery into cancerous and non-cancerous cells at very high concentrations 20, 24.

In the current study, we explored the longitudinal treatment efficacy of four complementary miRNAs (miRNA-100, miRNA-122, antimiRNA-10b, and antimiRNA-21) encapsulated in PLGA-PEG-NPs, administered by an US-guided and MB-mediated delivery approach in HCC xenografts. We used a human HCC cell line, HepG2, and a mouse HCC cell line, Hepa1-6, to determine the cellular and molecular effects of miRNA-100/miRNA-122/antimiRNA-10b/antimiRNA-21 loaded PLGA-PEG-NP treatments along with doxorubicin in vitro. HepG2 is a human HCC cell line that better mirrors the response to treatment in patients. However, HepG2 xenografts can only be established in immunocompromised mice. Therefore, we also evaluated in vitro treatment response in a mouse HCC cell line, Hepa1-6, which better mirrors the treatment response in immunocompetent mice with syngeneic HCC xenografts. For in vivo evaluation, we used tumor xenografts of HepG2 in immunocompromised mice, with ex vivo histology and quantification of delivered miRNAs as reference standards. Our results showed significant enhancement of doxorubicin treatment effect while using US-guided and MB-mediated targeted delivery of the four microRNAs in mouse models of HCC.

## Methods

### Materials

The materials used in this study and their sources are summarized in** Table 1.**

### Methods

**Figure 2:**
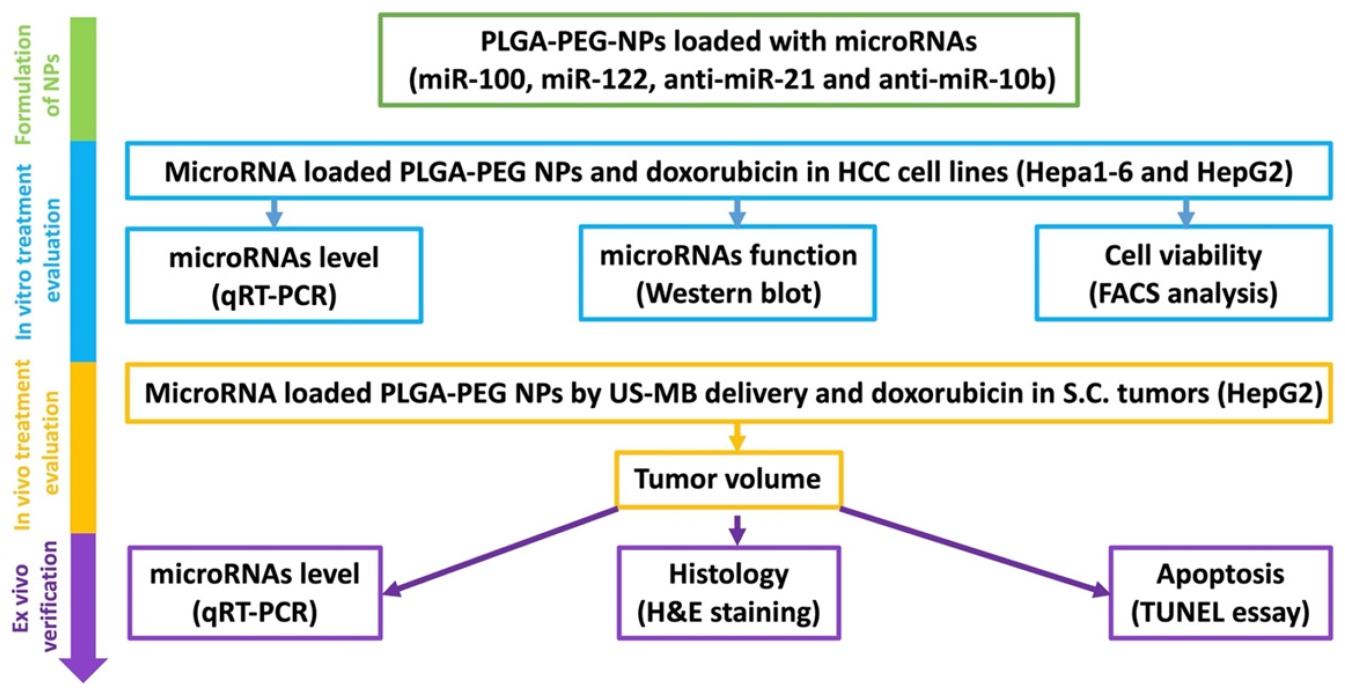
Schematic figure showing the overall *in vitro*, *in vivo* and *ex vivo* experimental design for evaluating the microRNA targeted HCC chemotherapy.

In the current study, we evaluated the treatment efficacy of four complementary miRNAs (miRNA-122, miRNA-100, miRNA-21, and miRNA-10b), which are crucial in HCC pathogenesis, drug resistance, and apoptosis. The treatment efficacy of the four miRNAs along with doxorubicin was tested in vitro (human and mouse HCC cell lines), in vivo (human HCC model of mice) and ex vivo (verification of in vivo outcomes) (**Figure 2**)*.*

### Cell culture

Human HepG2 and mouse Hepa1-6 HCC cells (ATCC, Manassas, VA) were grown in high glucose (4.5 g/L) Dulbecco's Modified Eagle's Medium (DMEM; Invitrogen, Carlsbad, CA) supplemented with fetal bovine serum (10%), penicillin (100 U/mL) and streptomycin (100 μg/mL). The cells were cultured by incubating at 37 °C in a humidified atmosphere of 5% CO_2_ and 95% air. The cells were periodically tested for STR profile for genomic integrity (Genetica, Burlington, NC), and for mycoplasma contamination using MycoAlert mycoplasma detection kit (Lonza, Hayward, CA).

### MicroRNA loaded nanoparticles synthesis and characterizations for their physicochemical properties

The four miRNAs loaded PLGA-*b*-PEG nanoparticles were synthesized as described previously 9, 10, 24, 25. Particle size and zeta potential of PLGA-*b*-PEG NPs were measured with dynamic light scattering (DLS) analysis by using a Zetasizer-90 (Malvern Instruments, Worcestershire, United Kingdom). Size measurement was performed at 25°C at a 90° scattering angle. The mean hydrodynamic diameter was determined by cumulant analysis. Zeta-potential measurements were performed using an aqueous dip cell in an automatic mode by Smoluchowski model. The concentration of PLGA-*b*-PEG nanoparticles was also estimated by Nanoparticle tracking analysis (NTA).

### The evaluation of endogenous miRNAs and intracellular uptake of delivered therapeutic miRNAs by qRT-PCR analysis

It has been shown that expression of endogenous miRNA-122 and miRNA-100 was significantly lower compared to control miRNA-10b, and expression of endogenous miRNA-21 was significantly higher compared to control miRNA-10b both in HepG2 and Hepa1-6 HCC cells before miRNA loaded PLGA-*b*-PEG-NPs delivery. After miRNA loaded PLGA-*b*-PEG-NPs delivery, therapeutic miRNA levels (miRNA-100, miRNA-122, antimiRNA-10b, and antimiRNA-21) were measured in both HepG2 and Hepa1-6 HCC cells by qRT-PCR analyses as described previously using Taqman qRT-PCR kit (Thermo Scientific, CA) for miRNA-21, miRNA-10b, miRNA-100, miRNA-122, and custom designed kits for antimiRNA-10b and antimiRNA-21 10.

### *In vitro* treatment response assessment following miRNAs loaded PLGA-*b*-PEG-NPs in combination with doxorubicin in HCC cells in culture

Treatment response was assessed with FACS analysis in both HepG2 and Hepa1-6 cells in the following conditions: 1) doxorubicin only at 0.25, 0.50, 1.00 or 1.50 µM; and 2) miRNA-loaded PLGA-*b*-PEG-NPs in various treatment conditions (single miRNA or in combinations). The following treatment conditions with miRNAs were tested (each miRNA at 10 picomoles): a): antimiRNA-21; b) antimiRNA-10b; c) miRNA-100; d) miRNA-122; e) antimiRNA-21 and antimiRNA-10b; f) antimiRNA-21, antimiRNA-10b and miRNA-100; g) antimiRNA-21, antimiRNA-10b and miRNA-122; h) antimiRNA-21, antimiRNA-10b, miRNA-100, and miRNA-122; i) antimiRNA-10b, miRNA-100 and miRNA-122; and j) negative control with nanoparticles only (**Table 2**).

### Western blot analysis

Effect of miRNA treatments on expression levels of anti-apoptotic and pro-apoptotic proteins in HCC cells was assessed by western blot analyses. Expression of anti-apoptotic proteins, including HoxD10, insulin like growth factor receptor-1 (IGF1R), CD320, and BCL2, and pro-apoptotic protein including programmed cell death 4 (PDCD4) and Bax were assessed to evaluate the efficacy of each of the above-mentioned treatment conditions. Western blotting was performed according to standard protocols as described previously 25, 26. The following treatment conditions were tested: a) antimiRNA-21; c) miRNA-100; d) miRNA-122; e) antimiRNA-21 and antimiRNA-10b; f) antimiRNA-21, antimiRNA-10b and miRNA-100; g) antimiRNA-21, antimiRNA-10b and miRNA-122; h) antimiRNA-21, antimiRNA-10b, miRNA-100, and miRNA-122; i) antimiRNA-10b, miRNA-100 and miRNA-122; and j) negative control with nanoparticles only.

α-Tubulin was used as a housekeeping gene protein loading control. Untreated HCC cells were used as controls. Briefly, HepG2 and Hepa1-6 cells plated in 12 well plates (1.0 x10^5^ cells/well) for 24 h were incubated with miRNAs of different treatment conditions for 48 h. Then, cells were lysed in RIPA lysis buffer. The proteins (100 μg/sample) resolved in 4-12% gradient gel was electroblotted onto nitrocellulose membrane. The membranes were incubated with primary antibodies for each target protein respectively and subsequently with peroxidase-conjugated goat anti-rabbit IgG secondary antibody. The blots were developed using Pierce ECL Western Blotting Substrate (Thermo Fisher Scientific, USA), imaged and quantified using an IVIS Lumina III In Vivo Imaging System (Caliper, Perkin Elmer).

### Apoptotic index on FACS

The effects of various treatment conditions in HCC cells (HepG2 and Hepa1-6) were assessed by measuring apoptotic index using propidium iodide (PI) staining based FACS analysis as described previously 25, 26. Briefly, at day 1, 10^5^ cells of HepG2 and Hepa1-6 were plated in 12 well plates and grown for 24 h. At day 2, cells were washed once with PBS, and then incubated with miRNAs loaded PLGA-*b*-PEG-NPs of each treatment condition for 24 h. At day 3, doxorubicin was added and incubated in the cells for another 48 h. At day 5, cells were collected and prepared for FACS analysis. At day 6, FACS analysis was performed for apoptotic index after staining with propidium iodide dye.

### Human HCC xenografts in mice

This in vivo study was approved by the Institutional Administrative Panel on Laboratory Animal Care. Female nude mice (Charles River; 6-8 weeks old, weighing 20-25 g; n = 48) were used for developing human HCC xenograft models for the study. Human HCC HepG2 cells were cultured in high glucose (4.5 g/L) DMEM supplemented with fetal bovine serum (10%), penicillin (100 U/mL) and streptomycin (100 μg/mL; Thermo Scientific, CA). The cells were trypsinized, and 5×10^6^ HepG2 cells were mixed with 50 μl normal saline and 50 μl Matrigel (BD Biosciences, San Jose, CA). The HepG2 cell suspension was injected subcutaneously on the cranial side of both lower hind limb regions of mice, respectively, to grow two tumors; one of which was used for intra-animal control in the same animal. Tumor size was measured 7 days after the injection of tumor cells using an electronic caliper. When the tumor reached 0.3 to 1.0 cm in maximum diameter (mean size, 0.63 cm), the tumor-bearing mice were used for the treatment evaluations.

### In vivo longitudinal assessment of tumor growth following repeated treatment cycles

MicroRNAs were i.v. administered at the concentration of 400 pmols each. Doxorubicin was i.p. administrated at a sub EC50 dose of 2.5 mg/kg (10 mg/kg is a standard EC dose). We used 63 tumors randomly assigned into 8 groups according to the different combinations of miRNAs: group 1 received i.v. injection of miRNA-122, antimiRNA-10b, and antimiRNA-21, and doxorubicin along with US treatment (in vitro treatment condition g; n=7); group 2 received miRNA-100, miRNA-122, antimiRNA-10b, and antimiRNA-21, and doxorubicin along with US treatment (in vitro treatment condition h; n=7); group 3 received miRNA-100, miRNA-122, and antimiRNA-10b, and doxorubicin along with US treatment (in vitro treatment condition i; n=7); group 4 received i.v. injection of miRNA-122, antimiRNA-10b and antimiRNA-21, and doxorubicin without US (n=7); group 5 received miRNA-100, miRNA-122, antimiRNA-10b, and antimiRNA-21, and doxorubicin without US (n=7); group 6 received miRNA-100, miRNA-122, and antimiRNA-10b, and doxorubicin without US (n=7); group 7 received doxorubicin only (n=8); and group 8 was a negative control and did not receive any treatment (n=13).

### Ultrasound-guided and microbubble-mediated delivery of therapeutic miRNAs in mice bearing HCC xenografts

Non-targeted MBs (MicroMarker; VisualSonics, Toronto, Canada) were used for both imaging and therapeutic purposes in this study. All mice were kept anesthetized with 2% isoflurane in room air at 2 L/min and placed on a heated stage in prone position. Necrosis and overall perfusion of tumors were evaluated in contrast mode imaging on a dedicated small animal US imaging system (Vevo 2100; VisualSonics) with a high frequency transducer (MS250; center frequency of 18 MHz). We manually injected 6.8 x 10^7^ MBs in 20 µl saline via tail vein within 2 seconds, and tumor perfusion and spontaneous necrosis were visualized in contrast mode imaging. Only the tumors without substantial spontaneous necrosis were used in this study.

After the tumor perfusion was confirmed, a Vantage 256 research US system (Verasonics, Redmond, WA) was configured for dual operation of US imaging guidance and therapy for the targeted miRNA delivery. A single high-frequency L11-5 transducer was used in this study for both imaging and therapeutic purposes, with the therapeutic pulse parameters optimized to obtain the most efficient drug delivery 27. MB cavitation induced by the focused US beam increases the vascular permeability in the targeted region and enhances the delivery of miRNA loaded PLGA-*b*-PEG-NPs from the vascular compartment into extracellular space, where the miRNA cargo is released with locally elevated dose. First, the tumor was located in the imaging mode and centered on the treatment region displayed on the screen. The system was then switched to therapeutic mode (center frequency, 7.8 MHz; US excitation type, focused; US cycles, 25; F-number, 2; Focal distance, 8 mm) and miRNA loaded NPs suspension (150 µL) was manually injected via tail vein within 15 seconds. In *in vivo* experiments, we used Verasonics Vantage 256 ultrasound research scanner with a single L11-5 linear transducer array operating at 7.8 MHz frequency with pulse repetition frequency (PRF) of 1 Hz and duty cycle of 25%. The *in vivo* US operating parameters optimized, validated, and reported in our earlier study were adopted as such in the present study to evaluate the therapeutic benefit of microRNA therapy. The operating parameters and acoustic pressure are summarized in **Table 3** 28.

Immediately after miRNA injection, MBs were continuously infused via tail vein at 6.75 x 10^7^ (50 µL)/min using an infusion pump (Kent Scientific, Torrington, CT). When the MB infusion reached to steady state (30 seconds after the start of infusion), 100 therapeutic pulses were applied (a duration of approximately 3 minutes).

### Ex vivo analysis of tumors

At day 18, all mice were humanely euthanized, and the tumor tissues were harvested for ex vivo analyses including hematoxylin eosin (H&E) staining, TUNEL staining for assessment of apoptosis, and qRT-PCR for the quantification of delivered miRNAs.

H&E staining: A portion of tumor tissues and various organs including heart, lung, liver, pancreas, spleen and kidneys were fixed in 10% neutral buffered formalin for 48 h, embedded in paraffin, sectioned into 4-µm-thick slices, and stained according to standard protocols 29, 30. Histological tissues were analyzed in random order for the treatment effect and toxicity.

TUNEL assay: A portion of tumor tissues frozen in optimal cutting temperature (OCT) compound (TissueTek; Torrance, CA) were sectioned into 10-µm-thick slices using a cryomicrotome (Leica CM1850, Wetzlar, Germany). A terminal deoxynucleotidyl transferase (TdT) nick-end labelling (TUNEL) assay was performed by using a Trevigen TACS 2 TdT-DAB (diaminobenzidine) in situ Apoptosis Detection Kit (Trevigen, Gaithersburg, MD, USA) according to the manufacturer's instructions. After staining, diaminobenzidine-staining positive apoptotic cells were assessed in the images acquired by scanning the slides with a digital slide scanner (NanoZoomer S60; Hamamatsu Corporation, Bridgewater, NJ).

RNA extraction and qRT-PCR analysis: We extracted total RNA from tumor tissues using a mirVana RNA extraction kit (Life technologies, Grand Island, NY) as per the manufacturer's protocol 26. We quantified the total RNA and checked it for purity using a Nanodrop spectrophotometer (Thermo scientific). After quantification 15 ng of total RNA equivalent was reverse transcribed using RT-primers (Life technologies) using a reverse transcription kit (Life technologies) to produce the corresponding cDNA. We carried out cDNA synthesis in a 15 μL reaction volume, and performed qRT-PCR using cDNA (5 ng of RNA equivalent) combined with TaqMan-PCR reagents (primer and probe mix). qRT-PCR was performed by setting 2 min incubation at 50 °C, followed by activation of the DNA polymerase at 95 °C for 10 min, 50 cycles of 95 °C for 15 s, and 60 °C for 60 s in a BioRad CFX qRT-PCR system (BioRad, Hercules, CA). The qRT-PCR reaction was carried out in a 20 μL reaction volume. We calculated the expression of miRNA using the 2 ^-ΔΔCT^ method.

### Statistical analysis

All continuous measurements were expressed as mean ± standard deviation. The two-sample Wilcoxon rank test was used to compare tumor volumes (normalized to the baseline value) between: 1) groups 1, 4, 7, and 8; 2) groups 2, 5, 7 and 8; 3) groups 3, 6, 7 and 8; and 4) groups 1-3. All statistical analyses were performed with statistical software (SPSS version 21; IBM Corporation, Endicott, NY). The significance level was set at 0.05.

## Results

### Size, zeta potential, and miRNA loading efficiency of PLGA-*b*-PEG nanoparticles

**Figure 3:**
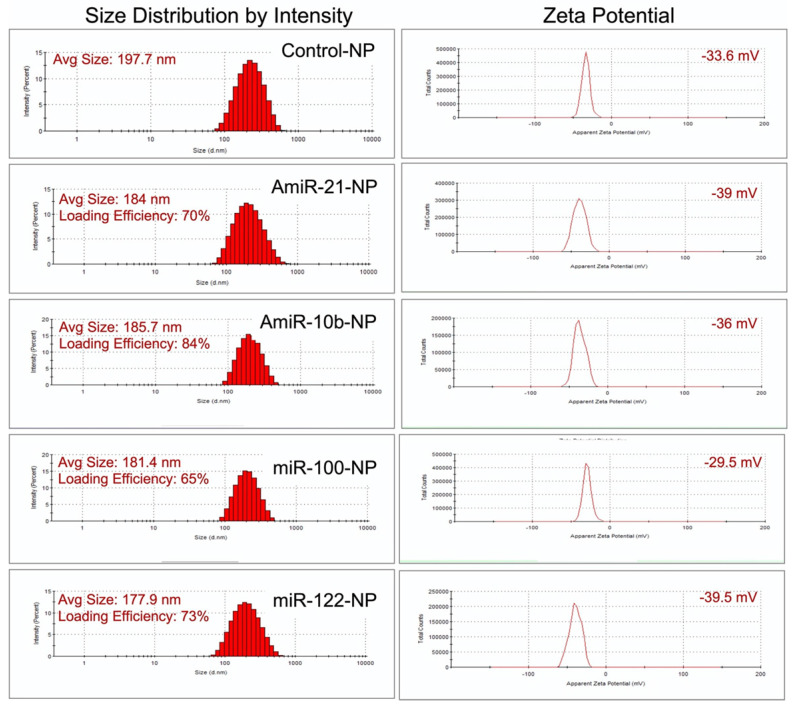
Size zeta potential and loading efficiency of miRNA-loaded nanoparticles.

We followed our well-optimized protocol for loading the four selective microRNAs that were used for therapeutic evaluations in HCC in this study 9, 10, 25. The size measurements using DLS revealed that the mean size of control plain NP was in the range of 197.7 nm, while the sizes of NPs loaded with miRNAs were the following: 184.0 nm for antimiRNA-21, 185.7 nm for antimiRNA-10b, 181.4 nm for miRNA-100, and 177.9 nm for miRNA-122. The mean zeta potential of control plain NP was -33.6 mV while the zeta potentials of NPs loaded with miRNAs were the following: -39.0 mV for antimiRNA-21, -36.0 mV for antimiRNA-10b, -29.5 mV for miRNA-100, and -39.5 mV for miRNA-122. MiRNA encapsulation efficiency in PLGA-*b*-PEG-NPs was 70% for antimiRNA-21, 84% for antimiRNA-10b, 65% for miRNA-100, and 73% for miRNA-122 (**Figure 3**). These results were consistent for various batches of NPs prepared during the entire study period.

### Therapeutic effects of complementary miRNAs on cell viability of HepG2 human HCC cells in the presence of doxorubicin

**Figure 4:**
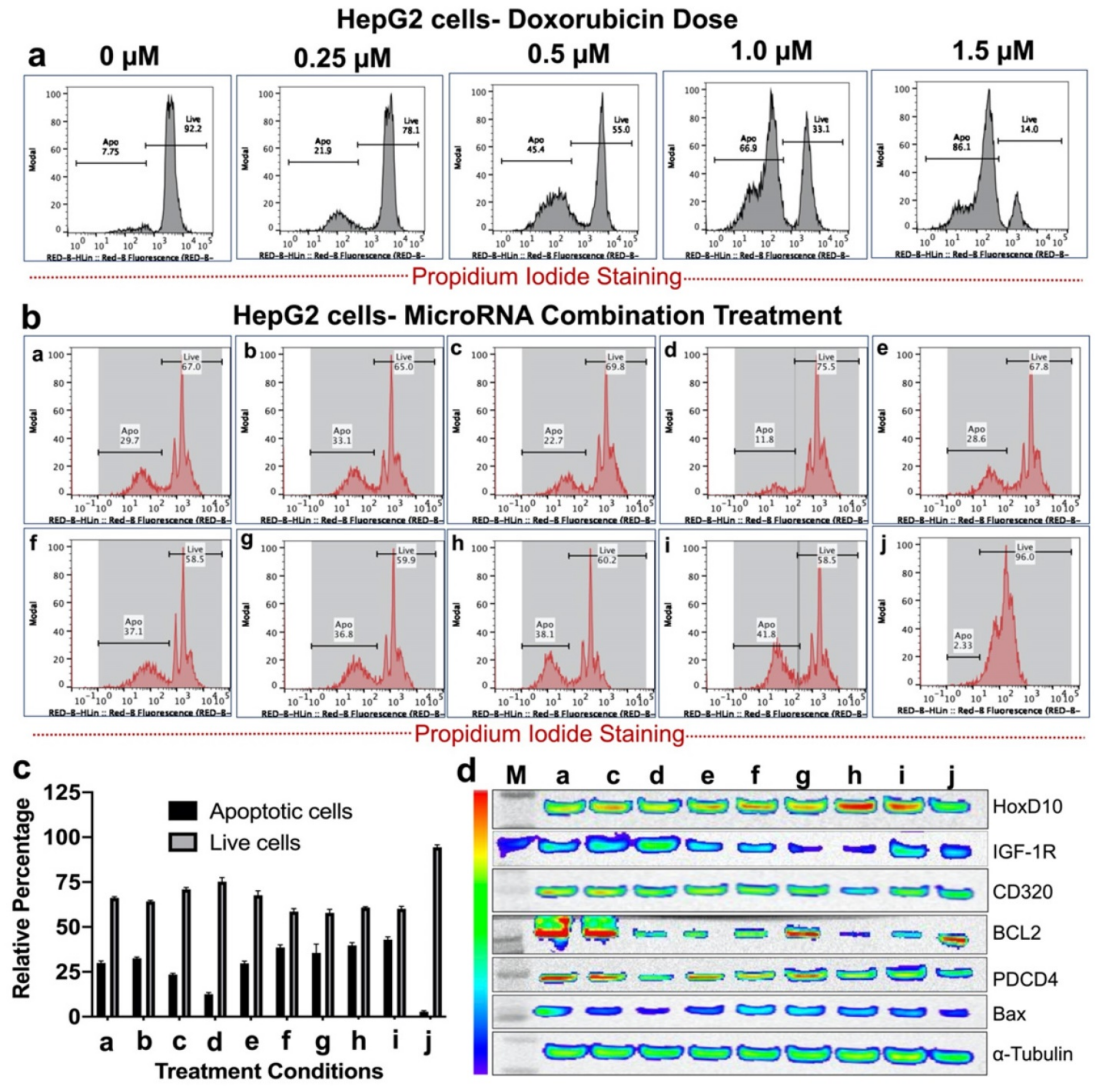
In vitro treatment evaluation of HepG2 human HCC cells to various combination of microRNAs in the presence of doxorubicin chemotherapy and the downstream functional effect by immunoblot analysis. **(a)** Doxorubicin dose mediated apoptotic evaluation, **(b)** MicroRNAs combination therapy evaluated in the presence of 0.25 μM doxorubicin using PI staining based FACS analysis for nine different miRNA combinations delivered by PLGA-*b*-PEG nanoparticles, **(c)** Quantitative graph showing the results of apoptotic and live cell populations measured by the FACS data shown in '**b**',** (d)** Western blot results showing strongest effect either on both downregulation of the target anti-apoptotic proteins HoxD10, IGF1R, CD320 and BCL2, or/and upregulation of the target pro-apoptotic proteins PDCD4 and Bax after various treatment conditions in HepG2 cells.

Single and various combinations of miRNAs shown in **Table 2** were used for treatment effect evaluations in HepG2 human HCC cells by measuring live and apoptotic populations using PI staining based FACS analysis (**Figure 4**). First, we tested the sensitivity of HepG2 cells to various concentrations of doxorubicin to identify the dose response. We used doxorubicin concentrations in the range of 0.25 μM to 2.0 μM. The cells analysed 48 hr after doxorubicin treatment showed a dose responsive apoptotic induction **(Figure 4a)**. We observed a minimum apoptotic induction of 21.2 ± 2.5% at 0.25 μM doxorubicin and a maximum level of more than 95% at 2.0 μM. We used 0.25 μM doxorubicin for various microRNA combination therapy since microRNAs sensitize cancer cells to chemotherapy rather than inducing apoptosis.

The results showed that for single miRNA treatment (antimiR-21, antimiR-10b, miRNA-100 or miRNA-122) in combination with 0.25 μM doxorubicin, the viability of HepG2 cells were slightly lower than 0.25 μM doxorubicin alone treatment for antimiR-21 and antimiR-10b. However, miRNA-100 and miRNA-122 showed no significant enhancement in the apoptotic populations. In contrast, various combinations of miRNAs treatment along with 0.25 μM doxorubicin, the viability of HepG2 cells were significantly affected in combination g (antimiRNA-21, antimiRNA-10b, and miRNA-122), h (antimiRNA-21, antimiRNA-10b, miR-100, and miRNA-122), and i (antimiRNA-10b, miRNA-122 and miRNA-100). Cellular viability of HepG2 cells was more than 96.0% for control condition without any treatment (**Figure 4b, c**).

### Therapeutic effects of complementary miRNAs on cell viability of Hepa1-6 mouse HCC cells in the presence of doxorubicin

**Figure 5:**
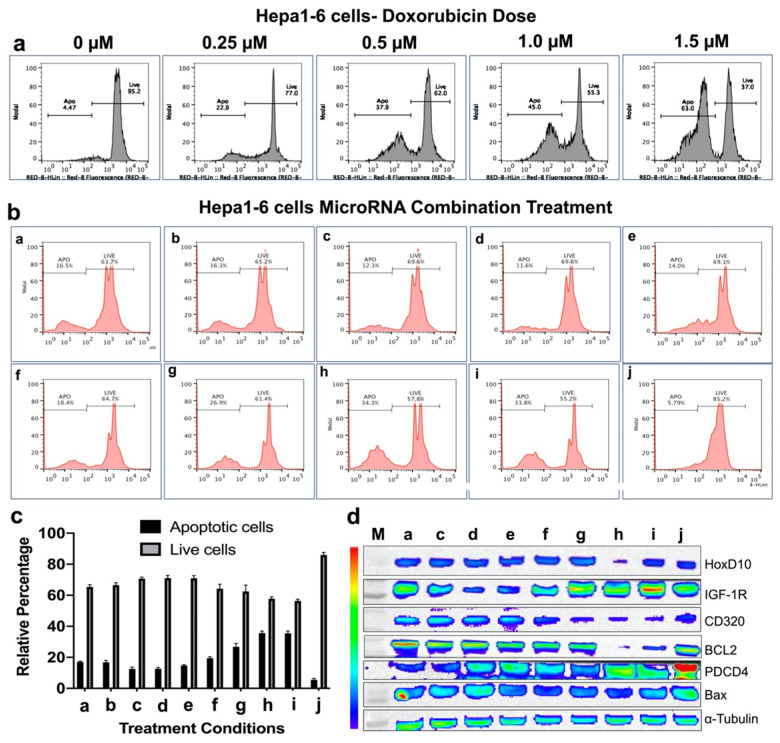
In vitro treatment evaluation of Hepa1-6 mouse HCC cells to various combination of microRNAs in the presence of doxorubicin chemotherapy and the downstream functional effect by immunoblot analysis. **(a)** Doxorubicin dose mediated apoptotic evaluation by PI staining based FACS analysis, **(b)** MicroRNAs combination therapy evaluated in the presence of 0.25 μM doxorubicin by PI staining based FACS analysis using nine different miRNA combinations delivered by PLGA-*b*-PEG nanoparticles,** (c)** Quantitative graph showing the results of apoptotic and live cell populations measured by the FACS data shown in '**b**',** (d)** Western blot results showing strongest effect either on both downregulation of the target anti-apoptotic proteins HoxD10, IGF1R, CD320 and BCL2, or/and upregulation of the target pro-apoptotic proteins PDCD4 and Bax after various treatment conditions in Hepa1-6 cells.

We used the same in vitro protocol for evaluating treatment effect in Hepa1-6 mouse HCC cells as we performed in the HepG2 cells. First, we evaluated the doxorubicin dose response. Similar to HepG2 cells, Hepa1-6 cells also showed comparable dose response trend to doxorubicin (**Figure 5a**). When we used 0.25 μM doxorubicin for microRNAs combination therapy, we observed no improvement in the apoptotic inductions for all the single microRNA therapies (antimiR-21, antimiR-10b, miRNA-100 or miRNA-122), and treatment condition e (antimiRNA-21 and antimiRNA-10b) and f (antimiRNA-21, antimiRNA-10b and miRNA-100). Whereas treatment conditions g (antimiRNA-21, antimiRNA-10b, and miRNA-122), h (antimiRNA-21, antimiRNA-10b miRNA-100 and miRNA-122), and i (antimiRNA-10b, miRNA-122 and miRNA-100) showed significant enhancement in apoptotic induction. Cell viability of Hepa1-6 cells was not affected in control condition where more than 85% of cells were alive after 72 h of incubation period in culture (**Figure 5b, c**).

Based on the in vitro treatment effects on both HCC cell lines, the three most promising treatment conditions, g (antimiRNA-21, antimiRNA-10b and miRNA-122), h (antimiRNA-21, antimiRNA-10b, miRNA-100, and miRNA-122) and i (antimiRNA-10b, miRNA-100, and miRNA-122), were chosen for subsequent in vivo treatment evaluations in a HepG2 xenograft model for the US-MB mediated targeted-delivery approach.

### Immunoblot analysis revealed microRNA mediated modulation of cellular targets in both HepG2 and Hepa1-6 HCC cells

After we evaluated enhanced therapeutic activity by microRNAs in combination with low dose doxorubicin, we tested altered expression of target proteins of microRNAs and pro- and anti-apoptotic genes by immunoblot analyses in both HepG2 and Hepa1-6 cells. Since doxorubicin tends to show no impact on these target genes except the apoptotic protein, we evaluated the combination treatment along with plain NP treated control condition in HepG2 and Hepa1-6 cells. The cells treated with the combination of microRNAs and 0.25 μM doxorubicin for 24 hrs and tested for different targets using the list of antibodies shown in the methods section. Western blot analyses showed strongest effect either on both downregulation of the target anti-apoptotic proteins HoxD10, IGF1R, CD320 and BCL2, or/and upregulation of the target pro-apoptotic proteins PDCD4 and Bax after treatments with treatment conditions (a-h) and doxorubicin in both Hepa1-6 and HepG2 cells, compared to negative control nanoparticle only treatment condition in treatment condition j (**Figure 4d and 5d**).

### Therapeutic effects of repeated treatments with complementary miRNAs in HepG2 human HCC tumor xenografts in vivo

**Figure 6:**
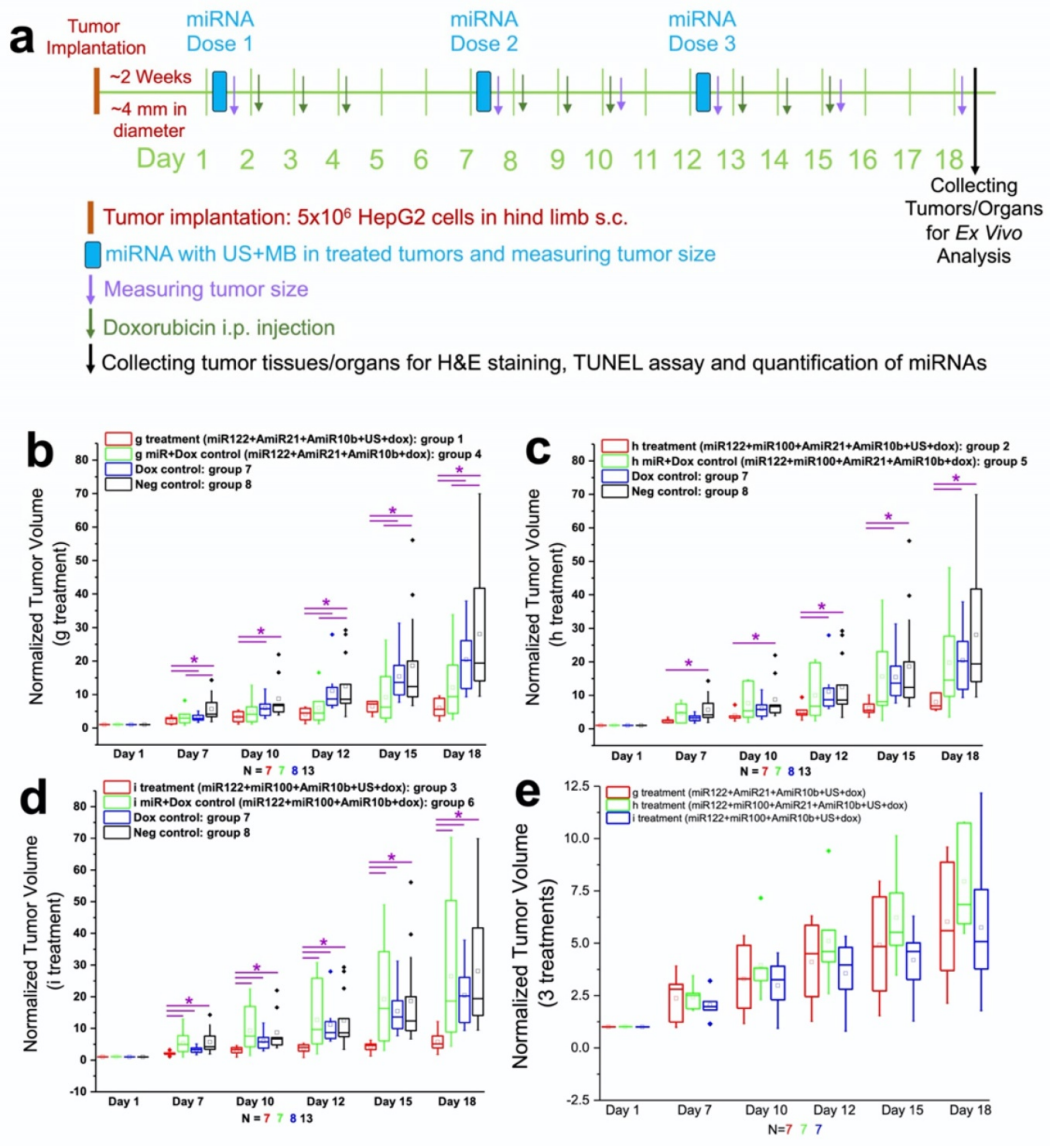
Antitumor effect of complementary miRNAs and doxorubicin co-treatment in the presence and absence of US-MB delivery in HepG2 human HCC xenografts *in vi*vo. **(a).** Schematic workflow summarizes the in vivo treatment conditions, therapy evaluation, and ex vivo analyses used for monitoring therapeutic outcome. Three repeated cycles of combination treatment were used on day 1, 7, and 12, for the delivery of miRNA-loaded NPs using an ultrasound-guided and microbubble-mediated delivery approach. On days 2, 3, 4, 8, 9, 10, 13, 14, and 15, the mice in groups 1-7 also received i.p. injection of low dose doxorubicin at 2.5 mg/kg. Tumor volume was calculated using the formula: volume = [length x width x height) × π /6. The tumor volumes were measured at the baseline (day 1) before treatment started, and 7, 10, 12, 15, and 18 days after treatment. To facilitate the comparisons between the tumor volumes in different groups, the tumor volumes measured at different time points in each animal were normalized to its own baseline value. Therefore, the normalized tumor volume value was 1.0 for each animal at the baseline. **(b)**. The results of the treatment condition g with miRNAs (antimiRNA-21, antimiRNA-10b, and miRNA-122) and doxorubicin, with (group 1) and without (group 4) US-MB treatment along with doxorubicin only and untreated control group monitored for tumor growth over 18 days. **(c)**. The results of the treatment condition h with miRNAs (antimiRNA-21, antimiRNA-10b, miRNA-100 and miRNA-122) and doxorubicin, with (group 2) and without (group 5) US-MB treatment along with doxorubicin only and untreated control group monitored for tumor growth over 18 days. **(d)**. The results of the condition group i with miRNAs (miRNA-122, miRNA-100 and antimiRNA-10b) and doxorubicin, with (group 3) and without (group 6) US-MB treatment along with doxorubicin only and untreated control group monitored for tumor growth over 18 days. **(e)**. The comparison between the three treatment groups (miRNA + doxorubicin + ultrasound) showing no statistical significance at day 7 to 18. Note that values were normalized and compared to day 1 value before the treatment. Each box in the plot represents the 25^th^ and 75^th^ quartiles, the line inside each box identifies the median and the whiskers indicate the 5^th^ and 95^th^ percentile of measurements excluding the outliers. ♦ represent outliers. * indicates P value < 0.05 between treated and control groups.

**Figure 6a** shows the schematic workflow of various treatment conditions used for the study. We used eight treatment groups which included in vitro treatment condition g with and without US-MB treatment (groups 1 and 4) plus doxorubicin, treatment condition h with and without US-MB treatment (groups 2 and 5) plus doxorubicin, treatment condition i with and without US-MB treatment (groups 3 and 6) plus doxorubicin, doxorubicin only treatment (group 7) and negative control group (group 8). In **Figures 6b-e**, we summarize the tumor growth measured in different treatment groups. The US-enhanced MB-mediated delivery of miRNAs along with doxorubicin enhanced the therapeutic effects in all treatment groups compared to the chemotherapy only and negative control groups.

In the treatment group 1 with miRNAs (g: antimiRNA-21, antimiRNA-10b, and miRNA-122), doxorubicin and US treatment, the tumor growth was significantly (P<0.05) slower than doxorubicin control group or negative control group without any treatment from day 7 to 18 (**Figure 6b**). Although the tumor growth in group 1 was slower than group 4 with miRNAs (antimiRNA-21, antimiRNA-10b, and miRNA-122) and doxorubicin treatment at all time points, there was no statistical significance between the two groups. This could probably be due to enhanced permeability and retention (EPR) effect and/or the enhanced delivery from the US-MB treatment of contralateral tumors. In addition, the tumor growth in group 4 was slower than doxorubicin control group 7 or negative control group 8 without any treatment at all time points (P < 0.05 for group 2 vs. group 8 at day 7, 12, 15, and 18) (**Figure 6b**).

In the treatment group 2 with miRNAs (h: antimiRNA-21, antimiRNA-10b, miRNA-122, and miRNA-100), doxorubicin and US treatment, the tumor growth was significantly (P<0.05) slower than negative control group without any treatment from day 7 to 18 (**Figure 6c**), and slower than doxorubicin-alone control group (P<0.05 for day 12, 15 and 18). Although the tumor growth in group 2 was slower than group 5 with miRNAs (antimiRNA-21, antimiRNA-10b, miRNA-122, and miRNA-100) and doxorubicin treatment at all time points, there was no statistical significance (P>0.05) between the two groups (**Figure 6c**).

In the treatment group 3 with miRNAs (i: antimiRNA-10b, miRNA-122, and miRNA-100) plus doxorubicin and US treatment, the tumor growth was significantly (P<0.05) slower than all the other control groups including group 6 with miRNAs (i: antimiRNA-10b, miRNA-122, and miRNA-100) and doxorubicin treatment but with no US treatment from day 7 to 18 (**Figure 6d**). When the therapeutic effects were compared between the three treatment groups 1-3, there was no statistical significance (P>0.05) between the any two groups from day 7 to 18 (**Figure 6e**).

### Ex vivo verification of therapeutic effects of repeated treatments with complementary miRNAs

**Figure 7:**
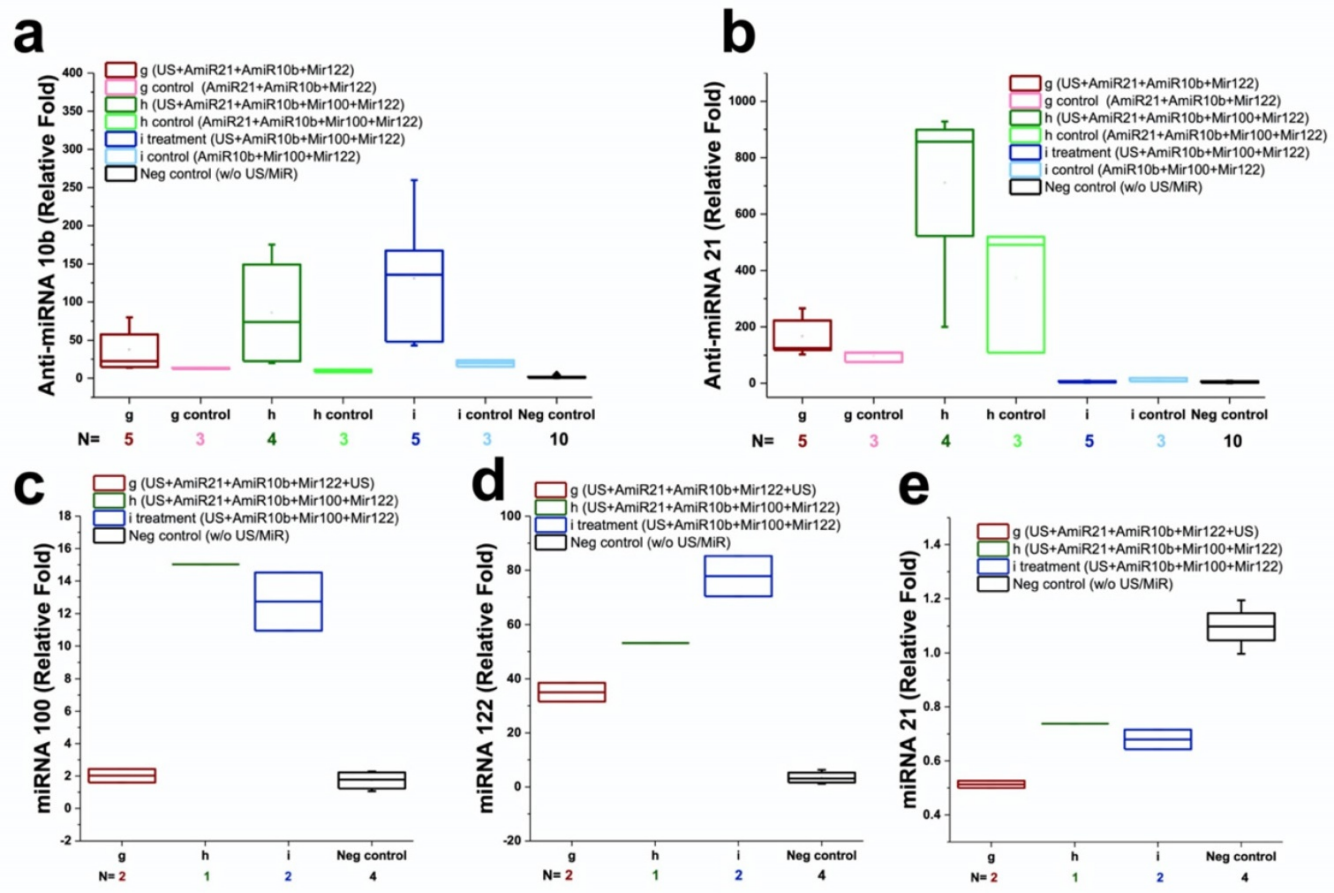
Quantitative RT-PCR results in human HepG2 HCC xenografts following intravenous injection of miR-100, miR-122, antimiR-10b, and antimiR-21-loaded PLGA-*b*-PEG-NPs and treated with ultrasound-guided and microbubble-mediated sonoporation. Note significant increases in the amount of delivered miRNAs were observed after 3 repeated treatment cycles in group 1-3 compared to controls.

We used quantitative RT-PCR to assess the amounts of miRNAs delivered into HCC xenografts after three sequential treatments ex vivo. **Figure 7** summarizes the results of miRNA levels in groups 1-8. Compared to negative control (group 8) without any treatment, the amounts of antimiRNA-21, antimiRNA-10b, miRNA-122, and miRNA-100 delivered in groups 1-3 were significantly (P<0.05) higher: 37.1 ± 16.1 -fold in group 1, 157.9 ± 76.0 -fold in group 2, and 5.2 ± 2.8 -fold in group 3 for antimiRNA-21, respectively; 23.6 ± 18.5 -fold in group 1, 53.6 ± 47.7 -fold in group 2, and 81.7 ± 56.4 -fold in group 3 for antimiRNA-10b, respectively; 1.2 ± 0.3 -fold in group 1, 8.8 ± 1.0 -fold in group 2, and 8.5 ± 1.5 -fold in group 3 for miRNA-100, respectively; 10.3 ± 1.4 -fold in group 1, 15.6 ± 1.0 -fold in group 2, and 25.1 ± 3.1 -fold in group 3 for miRNA-122, respectively **(Figure 7)**. Consistently, the level of endogenous miRNA-21 in groups 1-3 were significantly (P<0.05) lower compared to negative control (group 8) without any treatment: 56 ± 16% in group 1, 67 ± 0% in group 2, and 58 ± 5% in group 3. These results confirmed that the US treatment enhanced the delivery of miRNAs while also functionally altering the endogenous target miRNAs in cells.

**Figure 8:**
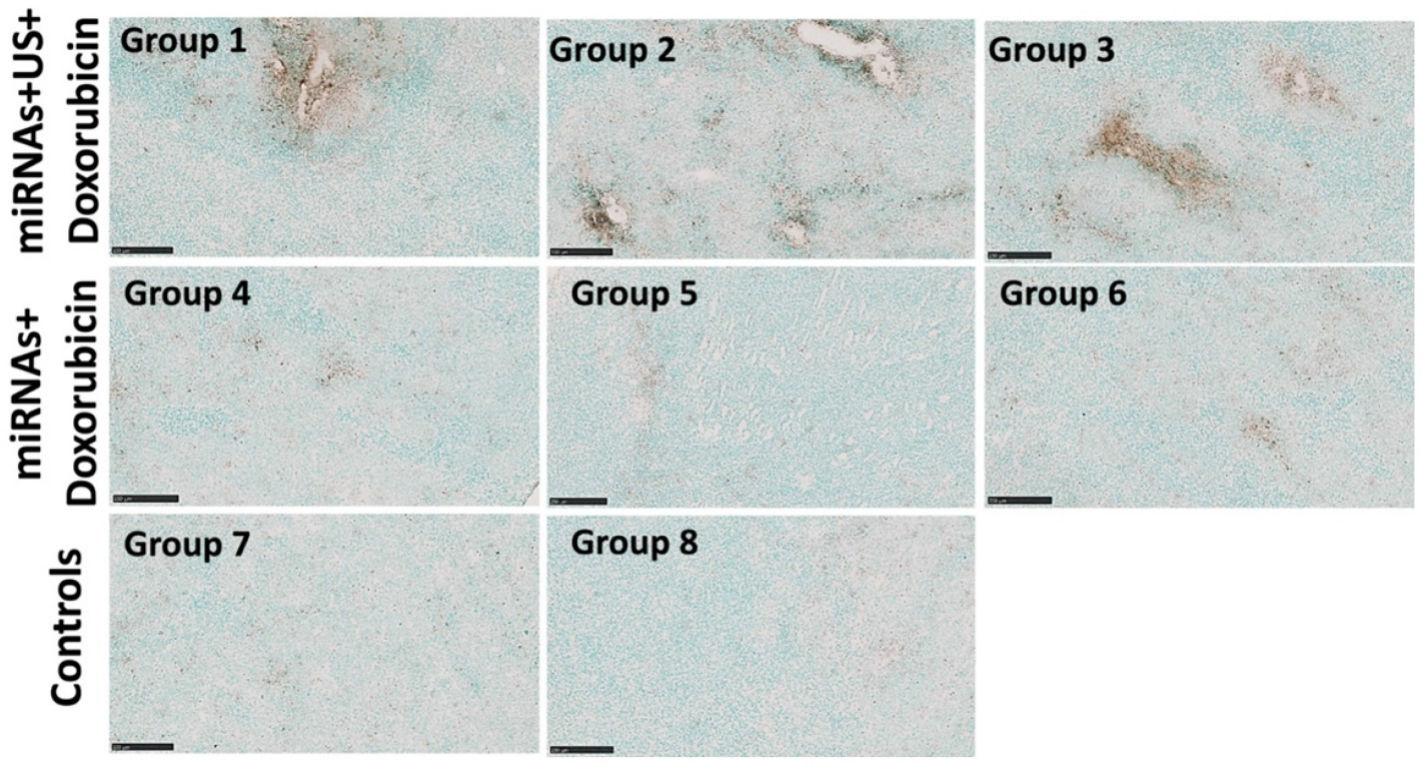
Representative TUNEL stained sections obtained from the animals with combination treatment show increased apoptosis (brown color) in treated tumors after 3 repeated treatment cycles compared to control tumors. Scale bars = 250 µm.

The therapeutic effects of repeated complementary miRNAs, doxorubicin along with US on apoptosis were assessed through the TUNEL assay. TUNEL assay revealed substantial enhancement of apoptosis with combinational treatments with miRNAs, doxorubicin and US in groups 1-3 compared to other control groups with miRNA and doxorubicin (groups 4-6 without US treatment), control group of doxorubicin only, and negative control group without any treatment (**Figure 8**).

**Figure 9:**
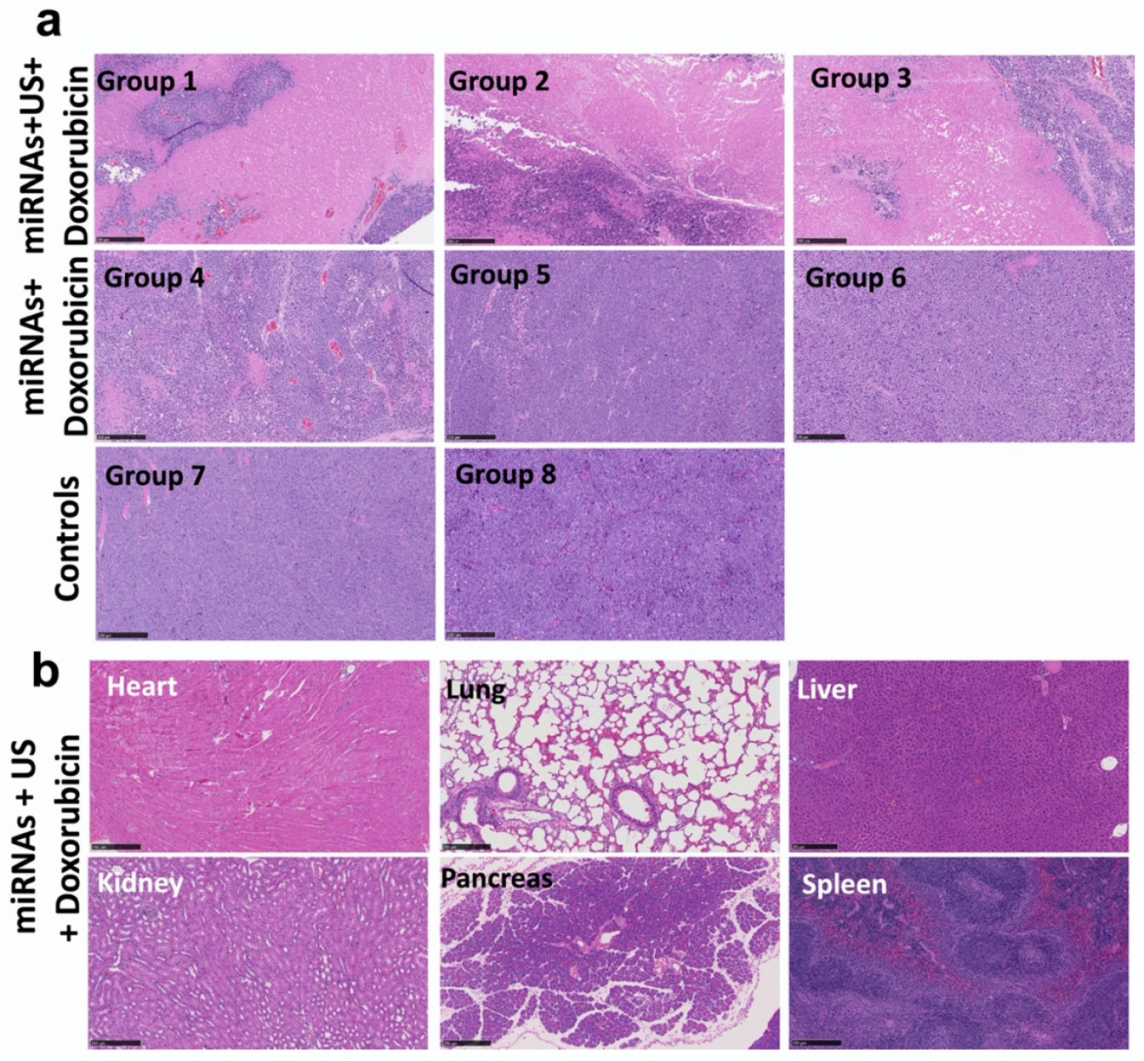
Histological analysis of tumors and different organs assessed for toxicity and treatment effect using Hematoxylin and Eosin (H&E) staining. **(a)** H&E stained sections show large tumor cell populations characterized by dense nuclear distribution with higher instance of actively dividing nuclear morphology in a representative animal of group 8. On the other hand, H&E stained sections of treated tumor demonstrate the enhanced necrosis in groups 1-3 with miRNA, doxorubicin and ultrasound.** (b)** H&E stained sections of various organ, including heart, lung, liver, pancreas, spleen and kidneys do not show the toxicity in the tumors treated with miRNA, doxorubicin and ultrasound. Scale bars = 250 µm.

The treatment effect in tumors and toxicity of the treatment in organs were evaluated by using H&E staining. The extensive necrosis was observed in the tumors of groups 1-3 treated with repeated complementary miRNAs, doxorubicin along with US (**Figure 9a**). No toxicity was found in the heart, lung, liver, pancreas, spleen and both kidneys in treated mice with tumors (**Figure 9b**).

## Discussion

In this study, we show that ultrasound-enhanced microbubble-mediated delivery of complementary miRNAs (miRNA-100, miRNA-122, antimiRNA-10b, and antimiRNA-21) targeting various oncogenic and tumor suppressor pathways along with chemotherapy of doxorubicin promoted the substantial increase of apoptosis of both human and mouse HCC cells in vitro, and significantly delayed the tumor growth in a mouse model of human HCC with a subtoxic dose of doxorubicin.

Ultrasound has a lot of advantages, such as relatively inexpensive cost, no ionizing radiation, portability, and worldwide availability 31. US-guided MB-mediated delivery approach enables the location-specific delivery of miRNAs and chemotherapeutic drugs and elevates their local effective dose at the tumor site, therefore leading to improve the efficiency of combination treatments 32-35. The majority of the human non-coding RNAs, including microRNAs that regulate gene expressions, are dysregulated in cancer and contribute to cancer initiation, metastasis, invasion, and drug resistance. MicroRNAs have been shown to be involved in the regulation of expression level of tumor-related genes and contribute to carcinogenesis, progression, and metastasis in HCC 5.

Although microRNAs are therapeutically promising molecules, their susceptibility to omnipresent RNase limits their delivery and functional efficiency. Thus, to overcome this drawback as elaborated in our earlier studies, we have used poly(ethylene glycol)-conjugated poly(lactic-co-glycolic acid) nanoparticles (PEG-PLGA-NPs) as a microRNA delivery vehicle to protect them from their premature degradation and to improve their antitumor effect for HCC by enhancing their bioavailability. Similar to other PEGylated nanoparticles, the PEG-PLGA nanoparticles adopted in this work may have some effect on the complement system, which is considered an integral component of the innate immune system 36-39. In such instances, an uncontrolled activation of the complement system can limit the delivery of nanoparticles to tumor tissues, while also bringing some detrimental effects in cancer therapy. In some of the recent works, researchers have demonstrated that, in contrast to carboxy-PEG-stabilized poly(lactic-co-glycolic acid) nanoparticles, surface camouflaging with appropriate combinations and optimal proportions of carboxy-PEG2000 and methoxy-PEG550 can largely suppress nanoparticle-mediated activation of the complement system through the lectin pathway. This is attributed to the ability of the short and rigid methoxy-PEG550 chains to laterally compress carboxy-PEG2000 molecules to become more stretched, and assume an extended and random coil configuration. Similarly, complement activation by PEGylated nanoparticles have also been indicated in other relevant literatures and in clinical trials 40, 41. It would be interesting to explore the potential of surface modification approaches, which would strengthen clinical potential of current work in the future.

In the current study, we evaluated the treatment efficacy of four complementary miRNAs which are important in HCC oncogenesis and drug resistance. We first evaluated the treatment effects of the four miRNAs alone and in various combinations along with doxorubicin in vitro to determine what combinations of the miRNAs were most effective in inducing apoptosis of the tumor cells in the presence of low dose doxorubicin (0.25 μM) in HCC cells. We assessed the effects of single and combined miRNA administration on cell proliferation and apoptosis in two different HCC cell lines: human HepG2 and mouse Hepa1-6 cells. Our results showed that combination treatment of 2-4 miRNAs could induce significantly higher apoptotic index in both HCC cell lines compared to any single miRNA treatment. According to the consistent results of apoptotic index in both HepG2 and Hepa1-6 cells, we chose three most effective combinations of miRNAs (miRNA-122/anitmiRNA-10b/antimiRNA-21, miRNA-100/miRNA-122/antimiRNA-10b/antimiRNA-21 and miRNA-100/miRNA-122/antimiRNA-10b) along with MB-mediated US treatment with doxorubicin administration for the in vivo evaluation.

For the in vivo delivery of miRNAs, we used a single L11-5 transducer for both therapy and imaging purposes, and optimized the therapeutic pulse parameters to obtain the most efficient drug delivery. US-guided MB-mediated delivery of miRNA-122 and antimiRNA-21 has been evaluated with a dual-transducer setting, with one transducer for imaging perfusion of tumor and the other for therapy purpose 9, 10. In the two-transducer configuration, it is time consuming to align and couple the two transducers and the resulting field-of-view is limited, which limits the two-transducer configuration in large animals (e.g., pig and dog) and application in humans. The advantage of the single-transducer configuration utilized in this study is that it enables the transducer to use its entire field-of-view to better visualize the tumor structure and perfusion, co-localizes the imaging and therapy planes without the need for calibration, and enables simultaneous imaging and treatment in a smaller and more manageable form factor. Although the spatial resolution of the imaging mode was not as high as with a dedicated small animal high-frequency US scanner, the transducer utilized in this study is a clinical-grade imaging transducer that was shown to be capable of emitting and maintaining therapeutic pulses while yielding sufficiently good imaging resolution of the tumor anatomy for aligning and locating the therapeutic plane and demonstrates that existing medical US technology can easily be adapted for therapeutic purposes.

We tested US-guided MB-mediated delivery of three promising combination of miRNAs along with doxorubicin in HepG2 xenografts in mice. Our results show that all the three combinational treatments significantly inhibited the tumor growth compared to negative control animals without any treatment as of 7 day after first cycle of the miRNA treatment. Our results are in concordance with previous studies of delivery of miRNA-122 and antimiRNA-21 9, 10. In addition, the three combinational treatments of miRNAs did not show any statistical difference between any 2 groups at any time point through day 18. Group 1 and 3 treatments both significantly inhibited tumor growth compared to the doxorubicin control group (group 7) from day 7 to 18, while such statistical significance between group 2 and doxorubicin alone treatment (group 7) was only reached at day 12 to 18. When we compared the miRNAs/US/doxorubicin groups of 1, 2 and 3 with miRNAs/doxorubicin groups 4, 5 and 6 without US respectively (group 1 vs 4, 2 vs 5 and 3 vs 6), we found that group 3 treatment significantly inhibited tumor growth compared to miRNAs/doxorubicin control of group 6 without US from day 7 to 18, but such statistical significance between group 1 vs 4, and groups 2 vs 5 was not reached from day 7 to 18. Moreover, the statistical significance was found between group 4 and 7 at day 7, 12, 15, and 18. Group 4 animals received miRNAs and doxorubicin, but not US treatment; group 7 animals received doxorubicin only. Such enhanced treatment efficacy of miRNAs even without US treatment was probably due to enhanced permeability and retention effect in tumors and/or the enhanced delivery from the US-MB treatment of contralateral tumors 42, 43.

The enhancement of chemotherapy efficacy by US-guided MB-mediated delivery of miRNAs was verified with increased expression levels of delivered miRNAs by qRT-PCR analyses. TUNEL assay and H&E staining also showed the significantly increased apoptosis/necrosis by this treatment strategy compared to doxorubicin only treatment. In addition, no toxicity was found in various organs of animals with this treatment strategy.

We acknowledge the following limitations in this study. First, we only monitored the tumor growth through 18 days after the treatment was started. The tumors in negative control group without any treatment reached to the maximum size allowed by the Institutional Administrative Panel on Laboratory Animal Care around day 18. Therefore, we had to euthanize all the animals for ex vivo analysis at day 18. Second, we used clinical US transducer for both imaging and therapy purposes in this study, and this transducer is not optimal for imaging tumors in small animals. In addition, the imaging system included basic B-mode image formation, but lacked the sophisticated image post-processing that is available in commercial US systems for small animal and medical imaging that generally yield substantial detailed anatomical information. Therefore, we had to wait to start the treatment until the tumor diameter reached to 4 mm (the minimal size at which the tumor can be accurately visualized and localized for therapy). With further image reconstruction enhancements and post-processing additions, we anticipate that earlier treatment can be applied when tumor diameter reaches up to 2-3 mm, and this can offer a better therapeutic evaluation window to monitor tumor growth over a longer period of time (longer than 18 days). The non-targeted microbubble used for contrast-enhanced ultrasound imaging in this study is purely a vascular contrast agent that gives the advantages of providing high sensitivity in observing hypervascularity and the real-time perfusion of the tumors. As the tumor grows, the necrotic region with poor vasculature develops, and it appears as a hypoechoic region in contrast enhanced ultrasound imaging. The necrosis associated hypovascularity also affects the US-MB mediated microRNA delivery in HCC. Such aspects are crucial in interpretation of tumor characteristics in a clinical setting as HCC is often diagnosed at the later stages. Possible problem-solving strategies around this limitation include a combination of US-MB mediated microRNA delivery along with a transient vascular promoting pharmacotherapy, as well as utilization of various hypervascularized tumor regions for the US based microRNA delivery 44. Third, the ex vivo verification with H&E staining, TUNEL assay and PCR for expression of delivered miRNAs was only performed at the end point, i.e., at day 18. The significant difference in tumor growth between treatment and control groups was found as of 7 days after the treatment was started. Therefore, the early ex vivo verification as of day 7 should be carried out in future studies. Fourth, although the combination treatment with miRNAs did not show any toxicity on H&E staining, US-guided MB-mediated delivery of nanoparticles may trigger an immune response in cancer 45. This warrants the further study to better understand the change of cytokines after US-guided MB-mediated delivery of miRNAs-loaded PLGA-*b*-PEG nanoparticles.

In conclusion, our results show that ultrasound-guided and microbubble-mediated delivery of miRNA-100/miRNA-122/antimiRNA-10b/antimiRNA-21 in combination with low dose doxorubicin induces substantial cell death and a significant decrease in tumor growth in a mouse model of human HCC, compared to doxorubicin only treatment. Our study further supports clinical development of this promising treatment platform for improving chemotherapy efficacy in patients with HCC.

## Figures and Tables

**Table 1:** Materials and Their Sources Used in this Study.

Materials	Vendor	Location
Primers for various PCR reactions	Customized	Institution (PAN)
MiR-100, miR-122, antimiR-21, antimiR-10b	Customized	Institution (PAN)
Media for cell culture	Thermo Fisher	Carlsbad, CA
RNA extraction kit (mirVana™ miRNA Isolation Kit)	Thermo Fisher	Waltham, MA
Reverse Transcriptase kit (mirVana™ miRNA Isolation Kit)	Thermo Fisher	Waltham, MA
Carboxy terminal Poly (lactic-co-glycolic acid)/-polyethylene glycol acid conjugate (PLGA-PEG) polymer	Poly SciTech	West Lafayette, IN
ToxinSensorTM Chromogenic LAL Endotoxin Assay Kit	Genscript	Piscataway, NJ
Contrast microbubbles (MicroMarker)	VisualSonics	Toronto, Canada
Primary antibodies and IgG anti-rabbit secondary antibody	Cell Signaling Technology	Danvers, MA
HepG2 and Hepa1-6 cells	American Type Culture Collection (ATCC)	Manassas, VA

**Table 2:** Treatment Conditions with Various Combinations of MiRNAs along with 0.25 µM Doxorubicin and Negative control without Any Treatment in HepG2 and Hepa1-6 Cells.

Various Treatment Conditions of MiRNAs along with 0.5 µM doxorubicin	HepG2 Cells	Hepa1-6 Cells
AntimiRNA-21	Condition a	Condition a
AntimiRNA-10b	b	b
MiRNA-100	c	c
MiRNA-122	d	d
AntimiRNA-21 and antimiRNA-10b	e	e
AntimiRNA-21, antimiRNA-10b and miRNA-100	f	f
AntimiRNA-21, antimiRNA-10b and miRNA-122	g	g
AntimiRNA-21, antimiRNA-10b, miRNA-100, and miRNA-122	h	h
AntimiRNA-10b, miRNA-100 and miRNA-122	i	i
Negative control (nanoparticle only treatment)	j	j

**Table 3:** Ultrasound operating parameters for L11-5 transducer used for in vivo experiments

Parameters	Value
I_SATA_ (W/cm^2^)	0.002
Frequency, MHz	7.8
US excitation type	Focused
No of US cycles	25
F/#	2
Focal distance, mm	8
No of areas/planes	11 areas
Treatment time (s)	100
PRF	1 Hz
PNP (MPa)	3.4

## Abbreviations

HCC: hepatocellular carcinoma
H&E: hematoxylin eosin
MB: microbubble
miRNA: microRNA
NP: nanoparticle
PCR: polymerase chain reaction
PEG: poly ethylene glycol
PLGA: poly lactic-co-glycolic acid
TUNEL: terminal deoxynucleotidyl transferase (TdT) nick-end labelling
US: ultrasound
UGMMTD: ultrasound-guided microbubble-mediated targeted delivery
